# Serotonin’s Role in Alcohol’s Effects on the Brain

**Published:** 1997

**Authors:** David M. Lovinger

**Affiliations:** David M. Lovinger, Ph.D., is an associate professor in the Department of Molecular Physiology and Biophysics, Vanderbilt University School of Medicine, Nashville, Tennessee

**Keywords:** acute AODE (alcohol and other drug effects), chronic AODE, serotonin receptors, brain, AODU (alcohol and other drug use) development, neurotransmission, synapse, neurotransmitters, serotonin uptake inhibitors, GABA, dopamine, receptor proteins, AOD abuse, AOD intoxication, AOD dependence, reinforcement, animal model, literature review

## Abstract

Serotonin is an important brain chemical that acts as a neurotransmitter to communicate information among nerve cells. Serotonin’s actions have been linked to alcohol’s effects on the brain and to alcohol abuse. Alcoholics and experimental animals that consume large quantities of alcohol show evidence of differences in brain serotonin levels compared with nonalcoholics. Both short- and long-term alcohol exposure also affect the serotonin receptors that convert the chemical signal produced by serotonin into functional changes in the signal-receiving cell. Drugs that act on these receptors alter alcohol consumption in both humans and animals. Serotonin, along with other neurotransmitters, also may contribute to alcohol’s intoxicating and rewarding effects, and abnormalities in the brain’s serotonin system appear to play an important role in the brain processes underlying alcohol abuse.

Neurotransmitters are chemicals that allow signal transmission, and thus communication, among nerve cells (i.e., neurons). One neurotransmitter used by many neurons throughout the brain is serotonin, also known as 5-hydroxytryptamine (5-HT). Serotonin released by the signal-emitting neuron subtly alters the function of the signal-receiving neurons in a process called neuromodulation. For example, in some neurons serotonin alters the rate at which the cells produce the electrical signals (i.e., action potentials) used for relaying information within the cells, whereas in other neurons it modulates the release of other neurotransmitters. (For more information on the mechanisms underlying signal transmission within and among neurons, see the article “The Principles of Nerve Cell Communication,” pp. 107–108.) Although serotonin’s effect on individual neurons can be rather modest, its overall effect on the neurons in a given brain area can substantially influence brain functions such as learning and memory, perception of the environment, mood states, and responses to alcohol and other drugs of abuse.

This article reviews serotonin’s functions in the brain and the consequences of acute and chronic alcohol consumption on serotonin-mediated (i.e., serotonergic) signal transmission. In addition, the article summarizes recent findings indicating that serotonin may play a pivotal role in the development of alcohol abuse.^1^

## Serotonin’s Functions in the Brain

Serotonin is produced in and released from neurons that originate within discrete regions, or nuclei, in the brain (Cooper et al. 1991). Many serotonergic neurons are located at the base of the brain in an area known as the raphe nucleus, which influences brain functions related to attention, emotion, and motivation. The axons of the neurons in the raphe nucleus extend, or project, throughout the brain to numerous regions with diverse functions. These brain regions include the amygdala, an area that plays an important role in the control of emotions, and the nucleus accumbens, a brain area involved in controlling the motivation to perform certain behaviors, including the abuse of alcohol and other drugs. In these brain regions, the axon endings of the serotonergic neurons secrete serotonin when activated. The neurotransmitter then traverses the small space separating the neurons from each other (i.e., the synaptic cleft) and binds to specialized docking molecules (i.e., receptors) on the recipient cell.

The binding of serotonin to its receptors initiates a series of biochemical events that converts the extracellular, chemical signal into an intracellular signal in the recipient cell. For example, the interaction of serotonin with one type of receptor stimulates the formation of small molecules (i.e., second messengers) within the cell. Second messengers interact with other proteins to activate various cellular functions, such as changes in the cell’s electrical activity or in the activity of certain genes (see figure). These changes can result either in the inhibition or the excitation of the signal-receiving neuron, depending on the cell affected. Through these mechanisms, serotonin can influence mood states; thinking patterns; and even behaviors, such as alcohol drinking.

Serotonin’s actions at the synapses normally are tightly regulated by proteins called serotonin transporters, which remove the neurotransmitter from the synaptic cleft after a short period of time by transporting it back into the signal-emitting cell. Consequently, serotonin can affect neighboring neurons only for a short period of time. Any interference with serotonin transporter function extends or diminishes the cells’ exposure to serotonin, thereby disrupting the exquisite timing of nerve signals within the brain. The net result of such disruptions is abnormal brain activity, which can lead to psychological problems or mental illness. One prominent example of a psychological disorder that appears to involve inappropriate serotonin use in the brain is depression (Baldessarini 1996); some of the most effective antidepressant medications act on the serotonin transporters to prolong the neurotransmitter’s activity.

**Figure:**
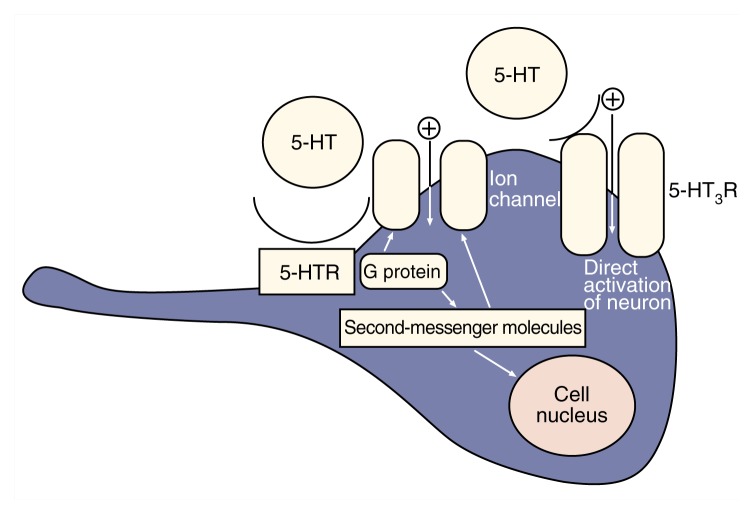
Activation of serotonin receptors (5-HTR) produces multiple effects on neurons. Serotonin (5-HT) can bind to receptors that activate proteins within the cell called G proteins. Activation of these proteins, in turn, affects ion channels in the cell membrane and induces the formation of signaling molecules (i.e., second-messenger molecules). Second messengers also can act on ion channels or travel to the nucleus to alter gene expression. Other serotonin-activated receptors (i.e., the 5-HT_3_ receptors) double as ion channels. Their activation directly excites neurons.

Researchers currently cannot directly measure serotonin concentrations in the human brain or within the synapses in laboratory animals. To gain information about serotonin levels in the brain, physicians and researchers have measured the concentrations of serotonin breakdown products generated after the neurotransmitter has been removed from the synapse (i.e., serotonin metabolites). The concentrations of these metabolites, which can be determined from samples of blood, urine, or the fluid that bathes the brain and spinal cord (i.e., cerebrospinal fluid [CSF]) (LeMarquand et al. 1994*a*; Pettinati 1996; Virkkunnen et al. 1995), provide an indirect measure of changes in the overall serotonin level in the brain.

## Acute Alcohol Effects on the Brain’s Serotonin System

Alcohol interacts with serotonergic synaptic transmission in the brain in several ways. Even single-episode (i.e., acute) alcohol exposure alters various aspects of serotonin’s synaptic functions. In humans, for example, the levels of serotonin metabolites in the urine and blood increase after a single drinking session, indicating increased serotonin release in the nervous system (LeMarquand et al. 1994*a*). This increase may reflect enhanced signal transmission at serotonergic synapses. Animal studies also have found that acute alcohol exposure elevates serotonin levels within the brain (LeMarquand et al. 1994*b*; McBride et al. 1993), suggesting either that more serotonin is released from the serotonergic axons or that the neurotransmitter is cleared more slowly from the synapses. For example, increased serotonin release after acute alcohol exposure has been observed in brain regions that control the consumption or use of numerous substances, including many drugs of abuse (McBride et al. 1993). Researchers currently are trying to determine the exact mechanisms underlying the alcohol-induced changes. For example, they are investigating whether the net increase in synaptic serotonin levels results from alcohol’s direct actions on molecules involved in serotonin release and uptake or from more indirect alcohol effects.

Alcohol also interferes with the function of serotonin receptors. Several types of these receptors exist, including the 5-HT_1A_, 5-HT_1B_, 5-HT_2_, and 5-HT_3_ receptors (see table). When activated by serotonin binding, the 5-HT_3_ receptor rapidly increases neuron activity by generating electrical signals (Lovinger and Peoples 1993). Acute alcohol exposure enhances the electrical signals generated by the 5-HT_3_ receptor. This change in receptor function likely results from alcohol’s direct action on the receptor protein or on molecules closely associated with the receptor in the cell membrane (Lovinger and Peoples 1993; Lovinger and Zhou 1994). Increased 5-HT_3_ receptor function probably causes excessive stimulation of neurons in brain regions receiving information from serotonergic neurons. As a result of this stimulation, the release of other neurotransmitters that play key roles in alcohol intoxication may be increased. The contribution of the 5-HT_3_ receptor to the effects of acute and chronic alcohol consumption is discussed later in this article.

The effects of acute alcohol consumption on serotonin receptors also have been investigated in so-called knockout mice, in whom certain genes (e.g., those coding for different serotonin receptors) have been experimentally inactivated so that the animals cannot produce the protein encoded by those genes. By studying knockout mice that lack a particular receptor, researchers can assess that receptor’s role in specific aspects of brain functioning and behavior, including responses to alcohol and alcohol consummatory behavior. For example, scientists have studied a strain of knockout mice lacking the 5-HT_1B_ receptor with respect to the effects of acute alcohol exposure (Crabbe et al. 1996). These animals exhibited reduced intoxication in response to a single dose of alcohol compared with normal mice, indicating that 5-HT_1B_ receptor activity produces some of alcohol’s intoxicating effects.

## Effects of Chronic Alcohol Exposure on Serotonergic Synaptic Transmission

Long-term, or chronic, alcohol exposure^2^ can lead to adaptive changes within brain cells. This process, also called tolerance development, presumably is a mechanism to reestablish normal cell function, or homeostasis, in response to continuous alcohol-induced alterations. For example, if alcohol exposure inhibits the function of a neurotransmitter receptor, the cells may attempt to compensate for continuous inhibition by increasing the receptor numbers or by altering the molecular makeup of receptors or cell membranes so that alcohol no longer inhibits receptor function. The 5-HT_2_ receptor appears to undergo such adaptive changes (Pandey et al. 1995). Thus, the number of 5-HT_2_ receptor molecules and the chemical signals produced by the activation of this receptor increase in laboratory animals that receive alcohol for several weeks.

Increased serotonin activity at the 5-HT_2_ receptor caused by chronic alcohol exposure also may contribute to the alcohol withdrawal syndrome—the pattern of behaviors occurring when alcohol is withheld after chronic use. For example, alcoholics frequently experience increased anxiety levels after cessation of drinking. This withdrawal symptom may involve enhanced serotonin activity at the 5-HT_2_ receptors: In animal models of alcohol withdrawal, drugs that blocked the activation of this receptor (i.e., 5-HT_2_ antagonists) prevented behavior indicative of increased anxiety (Lal et al. 1993).

The effects of chronic alcohol consumption also were investigated in the 5-HT_1B_ receptor knockout mice discussed in the previous section. Compared with normal mice, the knockout mice showed less evidence of tolerance to alcohol’s effects (Crabbe et al. 1996). Interestingly, the knockout mice also demonstrated increased aggressive behavior, even in the absence of alcohol consumption. A similar association between alcoholism and aggression exists in some alcoholics. Consequently, the 5-HT_1B_ receptor knockout mice may serve as a model for the alcoholism subtype that is characterized by an early age at onset and often is associated with impulsive violence and other behavioral disorders (Virkunnen et al. 1995).

## Serotonin’s Role in the Development of Alcohol Abuse

Two lines of evidence suggest that serotonin may be a key contributor to the brain dysfunction that leads to alcohol abuse: (1) analyses of serotonin levels in alcoholics and nonalcoholics and (2) studies of drugs that block serotonin receptors and serotonin transporters.

### Serotonin Levels in Alcoholics

The first line of evidence implicating serotonin in the development of alcohol abuse was the discovery of a relationship between alcoholism and the levels of serotonin metabolites in the urine and CSF of human alcoholics. For example, the concentrations of the first serotonin degradation product, 5-hydroxyindoleacetic acid, were lower in the CSF of alcoholics than in nonalcoholics of the same age and general health status (LeMarquand et al. 1994*a*; Pettinati 1996; Virkunnen et al. 1995), an observation suggesting that alcoholics may have reduced serotonin levels in the brain. Several mechanisms could account for such a decrease in brain serotonin levels. For example, the brain cells could produce less serotonin, release less serotonin into the synapse, or take more serotonin back up into the cells. Alternatively, the serotonin metabolite levels in alcoholics could be reduced, because less serotonin is broken down in the brain. To date, the exact mechanisms underlying the changes in serotonin-metabolite levels are still unknown.

Researchers currently are trying to determine whether alcoholics with abnormal serotonin metabolite levels have specific variations in the gene that codes for the enzyme tryptophan hydroxylase, which produces serotonin from other molecules in the cells. Several variants of the tryptophan hydroxylase gene exist; one variant appears to be particularly common in alcoholics with histories of aggression and suicidal tendencies (Virkkunen et al. 1995).

The relationship between serotonin levels and alcohol consumption also has been investigated in animal models of alcohol abuse. Some of the most intriguing findings have come from work on rats that were selectively bred for alcohol preference (P rats) or nonpreference (NP rats), based on the amounts of alcohol that they would drink when given a choice between alcoholic or nonalcoholic solutions.^3^

When the concentrations of different neurotransmitters were determined in various brain regions of these animals, the levels of serotonin and its metabolites were lower in P rat brains than in NP rat brains. The differences were particularly pronounced in the nucleus accumbens, a brain area thought to be involved in the rewarding effects of ethanol (LeMarquand et al. 1994*b*; McBride et al. 1995). Moreover, the P rats had fewer serotonergic neurons in the raphe nucleus compared with the NP rats (Zhou et al. 1994), a finding that could explain the reduced serotonin and serotonin-metabolite levels. The observation that P rats naturally have low serotonin levels supports the hypothesis that heavy drinking may partly represent an attempt to normalize serotonin levels in certain key brain regions, because acute alcohol consumption can elevate serotonin levels. Recent studies also have evaluated the numbers and properties of different serotonin receptors in P and NP rats. These studies found that P rats have fewer 5-HT_1A_ receptor molecules than do NP rats (DeVry 1995).

**Table:** Serotonin Receptor Subtypes and Their Potential Roles in the Development of Alcohol Abuse

Receptor Subtype	Potential Role in the Development of Alcohol Abuse
5-HT _1A_	May control consummatory behavior, including alcohol consumption
5-HT _1B_	May contribute to alcohol’s intoxicating effects May play a role in the development of tolerance to alcohol’s effects
5-HT_2_	May contribute to the development of alcohol withdrawal symptoms May play a role in alcohol’s rewarding effects
5-HT_3_	May regulate alcohol consumption May contribute to alcohol’s rewarding effects

### Effects of Serotonin Uptake Inhibitors

The second line of evidence implicating serotonin in the development of alcohol abuse stems from studies of compounds that interfere with the functions of the transporters that remove serotonin from the synapse. These agents also are called selective serotonin reuptake inhibitors (SSRI’s). One of these agents, fluoxetine (Prozac^®^), is used widely for treating mood disorders, such as depression (Baldessarini 1996). Experimental animals treated with this and related compounds exhibited reduced alcohol consumption (LeMarquand et al. 1994*b*; Pettinati 1996). Similarly, alcoholics taking fluoxetine drank less frequently and reduced their alcohol consumption during drinking sessions (LeMarquand et al. 1994*a*; Litten et al. 1996; Naranjo and Bremner 1994; Pettinati 1996). The alcoholics also reported less desire to drink and fewer pleasurable feelings after drinking. Fluoxetine reduces alcohol consumption in humans only moderately, however, and does not affect all alcoholics (Litten et al. 1996). Moreover, although increased serotonin levels at the synapses in the brain can moderate alcohol consumption, additional factors contribute to continued alcohol abuse. Consequently, SSRI’s cannot be recommended as the sole treatment for alcoholism.

Other drugs that affect serotonergic signal transmission also alter alcohol consumption in animals (LeMarquand et al. 1994*b*). For example, antagonists of the 5-HT_3_ and 5-HT_1A_ receptors reduced alcohol ingestion in rodents (Litten et al. 1996; Pettinati 1996; DeVry 1995). However, the 5-HT_1A_ receptor antagonists also altered food and water intake, suggesting that this receptor may modulate general consummatory behavior rather than specifically reduce the desire to drink alcohol. In humans, the 5-HT_3_ receptor antagonist ondansetron reduced total alcohol consumption and the desire to drink in alcoholics; as with the SSRI’s, however, this effect was relatively modest (Johnson et al. 1993; Pettinati 1996; Sellers et al. 1994).

More research is needed to determine how and under what drinking conditions alcohol consumption is affected by different serotonin receptor antagonists. In addition, researchers must investigate whether the effects of these drugs vary among subgroups of alcoholics (e.g., alcoholics with different drinking patterns or with co-occurring mental disorders). For example, recent evidence indicates that buspirone—an agent that binds to the 5-HT_1A_ receptor and which is used as an anxiety-reducing (i.e., anxiolytic) medication—also increases the time of abstinence from heavy drinking (Litten et al. 1996; Pettinati 1996). These findings suggest that buspirone may help reduce anxiety in alcoholics with anxiety disorders, thereby possibly improving their compliance with therapeutic regimens.

SSRI’s also are useful in treating anxiety, depression, and other mood disorders that result at least in part from dysfunctional serotonergic signal transmission in the brain (Baldessarini 1996). Many alcoholics suffer from these mood disorders. Accordingly, drugs that target serotonergic signal transmission may reduce alcohol consumption partly by improving the co-occurring psychiatric problems and thus eliminating the need for self-medication with alcohol. To some extent, however, the effects of SSRI’s on alcohol consumption appear to be unrelated to the medications’ antidepressant or anxiolytic effects (Naranjo and Kadlec 1991). The effects of SSRI’s and other serotonergic medications on alcohol abuse will be difficult to disentangle from their effects on co-occurring mental disorders. Nevertheless, the information currently available clearly indicates that serotonergic signal transmission plays an important role in alcohol abuse and therefore may yet be a target for therapies to reduce alcohol consumption.

## Interactions Between Serotonin and Other Neurotransmitters

Serotonin does not act alone within the brain. Instead, serotonergic neurons are parts of larger circuits of interconnected neurons that transmit information within and among brain regions. Many neurons within these circuits release neurotransmitters other than serotonin. Accordingly, some of the serotonin-mediated neuronal responses to alcohol may arise from interactions between serotonin and other neurotransmitters. These neurotransmitters also may be affected by alcohol. Two key neurotransmitters that interact with the serotonergic system are gamma-aminobutyric acid (GABA) and dopamine.

### Interactions With GABA

GABA is the major inhibitory neurotransmitter in the brain (Cooper et al. 1991)—that is, it tends to reduce the activity of the signal-receiving neuron. Many drugs that enhance GABA’s actions in the brain (e.g., the benzodiazepine Valium^®^) cause sedation and intoxication that resemble the effects of alcohol. In fact, alcohol may produce some of its sedative and intoxicating effects by enhancing GABA’s inhibitory function (Samson and Harris 1992). (For more information on alcohol’s effects on GABA-mediated signal transmission, see the article by Mihic and Harris, pp. 127–131.)

Serotonin may interact with GABA-mediated signal transmission by exciting the neurons that produce and secrete GABA (i.e., GABAergic neurons). For example, serotonin can increase the activity of GABAergic neurons in the hippocampal formation (Kawa 1994), a part of the brain that is important for memory formation and other cognitive functions. Consequently, alcohol’s effects on serotonin may alter the activity of GABAergic neurons in the hippocampal formation. These changes may disrupt cognition and possibly contribute to alcohol-induced memory loss and impaired judgment.

To activate hippocampal GABAergic neurons, serotonin binds to the 5-HT_3_ receptor. This receptor is present in many brain regions (Grant 1995) and may reside on GABAergic neurons. As discussed previously, alcohol increases the activity of this receptor. Increased 5-HT_3_ activity results in enhanced GABAergic activity, which, in turn, causes increased inhibition of neurons that receive signals from the GABA-ergic neurons. Other serotonin receptor types might act similarly on GABAergic neurons. Consequently, alcohol’s effects on these receptor subtypes also might influence GABAergic signal transmission in the brain.

### Interactions With Dopamine

The activation of serotonin receptors also modifies the activity of the neurotransmitter dopamine, which, like serotonin, modulates neuronal activity. The neurons that produce and secrete dopamine (i.e., dopaminergic neurons) reside at the base of the brain and communicate signals to brain regions involved in the rewarding effects of many drugs of abuse, including alcohol (Koob et al. 1994). For example, alcohol consumption induces a dopamine surge in the brain, which is thought to signal to the brain the importance of this action, thereby indicating that alcohol consumption is an action that should be continued. Such a response to alcohol ingestion easily could contribute to the development of an addiction to alcohol, because these brain responses would tend to reinforce alcohol drinking and thus increase consumption. (For more information on dopamine-mediated signal transmission, see the article by Di Chiara, pp. 108–114.)

Serotonin can alter dopaminergic signal transmission in several ways. For example, by interacting with the 5-HT_2_ receptor, serotonin stimulates the activity of dopaminergic neurons in a brain region called the ventral tegmental area (VTA), thereby enhancing an alcohol-induced increase in the activity of these neurons (Brodie et al. 1995) and causing increased dopamine release (Campbell et al. 1996). The dopaminergic neurons in the VTA are connected to the brain areas thought to mediate rewarding effects. Thus, the serotonin-dependent activation of these neurons could reinforce alcohol-drinking behavior. This scenario suggests that serotonin, through its interaction with the dopaminergic system, may play a pivotal role in producing alcohol’s rewarding effects.

Serotonin also interacts with dopaminergic signal transmission through the 5-HT_3_ receptor, which helps control dopamine release in the areas reached by VTA neurons, most notably the nucleus accumbens. Serotonin release in these brain regions can stimulate dopamine release, presumably by activating 5-HT_3_ receptors located on the endings of dopaminergic neurons (Campbell and McBride 1995; Grant 1995). Consequently, an alcohol-induced increase in 5-HT_3_ receptor activity would enhance dopamine release in these brain regions, thereby contributing to alcohol’s rewarding effects. This hypothesis is supported by the results of studies in animal models (Campbell and McBride 1995; Grant 1995; Wozniak et al. 1990), which also found that 5-HT_3_ receptor antagonists interfered with the serotonin-induced dopamine release in the brain’s reward systems. These findings may help explain the antagonists’ ability to reduce drinking behavior.

These examples demonstrate that serotonin interacts with other neurotransmitters in several ways to promote alcohol’s intoxicating and rewarding effects. Serotonin also may interact with additional neurotransmitters that have been found to contribute to alcohol’s effects on the brain.

## Summary

Serotonin plays an important role in mediating alcohol’s effects on the brain. Alcohol exposure alters several aspects of serotonergic signal transmission in the brain. For example, alcohol modulates the serotonin levels in the synapses and modifies the activities of specific serotonin receptor proteins. Abnormal serotonin levels within synapses may contribute to the development of alcohol abuse, because some studies have found that the levels of chemical markers representing serotonin levels in the brain are reduced in alcoholic humans and chronically alcohol-consuming animals. Moreover, SSRI’s and receptor antagonists can reduce alcohol consumption in humans and animals, although these agents are only moderately effective in treating alcohol abuse.

Serotonin is not the only neurotransmitter whose actions are affected by alcohol, however, and many of alcohol’s effects on the brain probably arise from changes in the interactions between serotonin and other important neurotransmitters. Thus, one approach researchers currently are pursuing to develop better therapeutic strategies for reducing alcohol consumption focuses on altering key components of the brain’s serotonin system.

## Footnotes

1 Throughout this article, the term “alcohol abuse” is used to describe any type of alcohol consumption that causes social, psychological, or physical problems for the drinker. Thus, the term encompasses the clinical diagnoses of alcohol abuse and alcohol dependence as defined by the American Psychiatric Association.

2 Generally, alcohol exposure for more than 1 day is considered chronic, because this time period exceeds the usual duration of a single session of drinking and intoxication. In animal experiments, however, chronic exposure periods can last several months, and humans often will drink continuously for months or years at a time.

3 By breeding rats with similar alcohol-consumption patterns (e.g., high consumption or low consumption) with each other for several generations, researchers created two strains with distinctly different preferences for alcohol.
